# Intravenous artesunate plus Artemisnin based Combination Therapy (ACT) or intravenous quinine plus ACT for treatment of severe malaria in Ugandan children: a randomized controlled clinical trial

**DOI:** 10.1186/s12879-017-2924-5

**Published:** 2017-12-28

**Authors:** Pauline Byakika-Kibwika, Jane Achan, Mohammed Lamorde, Carine Karera-Gonahasa, Agnes N. Kiragga, Harriet Mayanja-Kizza, Noah Kiwanuka, Sam Nsobya, Ambrose O. Talisuna, Concepta Merry

**Affiliations:** 1 Department of Medicine, College of Health Sciences, Makerere University, P. O. Box, 7072 Kampala, Uganda; 2 Infectious Diseases Institute, Kampala, Uganda; 3 Medical Research Council Unit, Serekunda, The Gambia; 4 School of Public Health, Makerere University, Kampala, Uganda; 5 Department of Pathology, Makerere University, Kampala, Uganda; 6 University of Oxford-KEMRI-Wellcome Trust Programme, Nairobi, Kenya; 7 Trinity College Dublin, Dublin, Ireland

**Keywords:** Artesunate, Quinine, Artemisinin combination therapy, Severe malaria, Re-infection

## Abstract

**Background:**

Severe malaria is a medical emergency associated with high mortality. Adequate treatment requires initial parenteral therapy for fast parasite clearance followed by longer acting oral antimalarial drugs for cure and prevention of recrudescence.

**Methods:**

In a randomized controlled clinical trial, we evaluated the 42-day parasitological outcomes of severe malaria treatment with intravenous artesunate (AS) or intravenous quinine (QNN) followed by oral artemisinin based combination therapy (ACT) in children living in a high malaria transmission setting in Eastern Uganda.

**Results:**

We enrolled 300 participants and all were included in the intention to treat analysis. Baseline characteristics were similar across treatment arms. The median and interquartile range for number of days from baseline to parasite clearance was significantly lower among participants who received intravenous AS (2 (1–2) vs 3 (2–3), *P* < 0.001). Overall, 63.3% (178/281) of the participants had unadjusted parasitological treatment failure over the 42-day follow-up period. Molecular genotyping to distinguish re-infection from recrudescence was performed in a sample of 127 of the 178 participants, of whom majority 93 (73.2%) had re-infection and 34 (26.8%) had recrudescence. The 42 day risk of recrudescence did not differ with ACT administered. Adverse events were of mild to moderate severity and consistent with malaria symptoms.

**Conclusion:**

In this high transmission setting, we observed adequate initial treatment outcomes followed by very high rates of malaria re-infection post severe malaria treatment. The impact of recurrent antimalarial treatment on the long term efficacy of antimalarial regimens needs to be investigated and surveillance mechanisms for resistance markers established since recurrent malaria infections are likely to be exposed to sub-therapeutic drug concentrations. More strategies for prevention of recurrent malaria infections in the most at risk populations are needed.

**Trial registration:**

The study was registered with the Pan African Clinical Trial Registry (PACTR201110000321348).

## Background

Malaria is a major cause of morbidity and mortality worldwide. In 2015, the WHO estimates that there were 214 million cases of malaria and 438,000 deaths worldwide, with 90% of deaths occurring in Africa [1]. The vast majority of severe malaria cases and deaths occur among children younger than 5 years of age and are caused by *Plasmodium falciparum* [2]. Severe malaria is a life threatening emergency, which usually causes death in the first 24–48 h following hospitalization. Mortality reduces from almost 100% to 10–20% following prompt and effective treatment [2]. The primary objective of severe malaria treatment is to prevent death while secondary objectives include prevention of disability and recrudescence. Treatment includes initial parenteral therapy for fast parasite clearance for a minimum of 24 h and when the patient is able to tolerate oral therapy, an effective oral artemisinin based combination therapy (ACT) is administered to achieve complete parasite clearance and to prevent recrudescence.

Two previous multicentre randomized trials demonstrated superiority of intravenous artesunate (AS) over intravenous quinine (QNN) for treatment of severe malaria in Africa and Asia [3–7]. Intravenous AS was associated with significant reduction in mortality of 22.5% in African children and 38.6% in Asian children and adults. These data led to the recommendation by the World Health Organization (WHO) for intravenous AS to replace intravenous QNN as the treatment of choice for severe *P. falciparum* malaria worldwide. Uganda adopted the policy to use intravenous AS in 2013 with intravenous QNN recommended as an alternative. To date, intravenous QNN is still widely prescribed due to inconsistent availability and frequent stock-outs of intravenous AS in health facilities [8].

The Ugandan malaria treatment policy recommends artemether-lumefantrine (AL) and dihydroartemisinin-piperaquine (DP) as the first and second line ACTs for follow-on therapy. Previous studies demonstrated excellent efficacy and effectiveness of both AL and DP for treatment of uncomplicated malaria [9–11] with the additional advantage of post treatment prophylactic effect of up to 28 days for AL and 35 days for DP [12]. Data on the parasitological outcomes following treatment of severe malaria with parenteral antimalarial drugs followed by oral ACTs are limited. Patients with severe malaria may survive death with the initial parenteral antimalarial regimen but may suffer morbidity due to recurrent parasitaemia [13] from reinfection and recrudescence. We evaluated the 42-day parasitological treatment outcomes and safety of ACT (AL or DP) following treatment of severe malaria among patients attending Tororo District Hospital in Eastern Uganda.

## Methods

### Study design, site and population

The study was a randomized single blind clinical trial conducted in Tororo District Hospital situated in Eastern Uganda, an area with perennial malaria transmission and an annual entomological inoculation rate estimated to be 310 infective bites per person per year [14].

Consecutive patients presenting to the hospital with symptoms and signs of severe malaria and a microscopy positive thick blood film for malaria parasites were referred to the study physicians for further assessment for study eligibility. Patients were enrolled if they fulfilled the following inclusion criteria: age 6 months and above, with history of fever in the last 24 h or axillary temperature > 37.5 °C, a diagnosis of severe malaria and whose parent or guardian provided written informed consent. Severe malaria was defined as presence or history of fever plus a positive blood film for *P.falciparum* malaria with at least one of the following symptoms and signs; repeated convulsions (more than 2 in 24 h), impaired consciousness (Glasgow Coma Score < 11 for adults and Blantyre Coma Score < 3 for children), coma, hyperpyrexia (axillary temperature >/= 40 °C), respiratory distress (acidotic breathing), inability to tolerate oral therapy and vomiting all oral intake, circulatory collapse or shock (systolic Blood Pressure < 80 mmHg in adults and <50 mmHg in children), spontaneous bleeding, haemoglobinuria (tea colored urine), jaundice, prostration (extreme weakness with inability to sit, stand or walk without assistance), severe normocytic anemia (hemoglobin <5 g/dL), hypoglycemia (blood sugar <2.2 mmol/l or 40 mg/dL), renal impairment (serum creatinine >265 μmol/l), and hyperparasitaemia (>10%). Patients were excluded if they had obvious concomitant febrile illness, history of allergy to any of the study drugs, were resident more than 20 km from the hospital and therefore could not return for follow-up visits or if they had history of receipt of an effective antimalarial drug within 72 hours before presenting to hospital. The last exclusion criterion was modified to exclude patients with history of receipt of an effective antimalarial drug within 24 hours before presenting to hospital due to large numbers of patients reporting self medication with antimalarial drugs prior to presenting to the hospital, thus affecting recruitment.

### Study procedures

#### Randomization

A 2-stage block randomization procedure was performed by an independent statistician using the R computer software in blocks of 20. Participants were first randomized to intravenous AS or intravenous QNN then to AL or DP. Computer-generated randomization codes were enclosed in sequentially numbered opaque sealed envelopes containing treatment allocation. After meeting study eligibility criteria, the study nurse assigned the next envelope to the participant, opened the envelope and assigned treatment allocation. Treatment was immediately initiated.

### Treatment

Intravenous AS (Guilin Pharmaceutical Factory, Guangxi, China) was administered as 2.4 mg/kg at start of treatment, repeated at 12 and 24 h and every 24 h till the switch to oral therapy. Each 60 mg vial of artesunic acid was dissolved in 1 mL of 5% sodium bicarbonate (provided in the pack) and mixed with 5 mL of 5% dextrose, then injected as a slow bolus into an indwelling intravenous cannula. Intravenous QNN dihydrochloride (Rotex, Trittau, Germany) was administered as 10 mg/kg body weight in 5% dextrose (10 ml/kg) through an indwelling catheter over 4 h and repeated 8 hourly till the switch to oral therapy.

Intravenous antimalarial therapy was administered for a minimum of 24 h and when participants could tolerate oral therapy, they were given a full course of the oral ACT assigned. Oral AL (Coartem, Novartis, 20 mg artemether/120 mg lumefantrine tablets) was administered according to body weight as; one (5–14 kg), two (15–24 kg), three (25–34 kg) and four (>35 kg) tablets given twice daily for 3 days. Parents and care takers were given instructions to administer the second dose of AL 8 hours after the first then every morning and evening to complete a 3 day course and all doses to be administered after food or a cup of milk. Oral DP (Eurartesim, Sigma-Tau, dihydroartemisinin (DHA) 40 mg + piperaquine (PQP) 320 mg tablets) was administered targeting a total of 6.4 and 51.2 mg/kg of dihydroartemisinin and piperaquine, respectively, given in three equally divided doses to the nearest quarter tablet. We used a pill cutter to ensure that the tablet fractions were as close to the nearest quarter tablet as possible.

All participants received paracetamol in a dose of 15 mg/kg at 8 hourly intervals. Adjunctive and supportive treatment such as diazepam for convulsions and dextrose for hypoglycemia was given in accordance with the Uganda Ministry of Health guidelines. All caretakers were given counseling on adherence to drugs, follow-up visits and potential drug side effects by the study nurse. They were instructed to observe the participants for 30 min after drug administration and if vomiting occurred they were to administer another dose. If vomiting occurred repeatedly (> 3 times) they were to bring back the participant to the study clinic for evaluation and treatment plus collection of additional doses of drugs.

### Follow-up

Serial blood smears were performed at 0, 1, 2, 4, 6, 8, 10, 12, 16, 20, 24 h post start of intravenous drugs followed by every 6 h until 6 h after parasite clearance. Participants were discharged from hospital when the blood smear was negative for malaria parasites and were followed up for 42 days to ascertain parasitological outcomes and monitor for adverse events. They were brought back to the clinic for follow up assessment on days 1, 3, 7, 14, 21, 28, 35, 42 post the commencement of ACT administration, and any unscheduled day if the participant felt unwell. Follow-up assessment on each of these days consisted of medical history, physical examination and a finger prick to collect blood on slides for thick blood film for malaria diagnosis and on filter paper for genotyping. Participants with positive malaria films were reassessed for severity and treated accordingly. Participants who returned with severe malaria were re-admitted and treated with intravenous AS plus AL. Those who returned with uncomplicated malaria were evaluated for treatment failure and treated according to national guidelines with DP if the blood smear was positive before day 14 and AL if it was positive after day 14 post commencement of ACT. Participants were discontinued from study follow up if they could not continue with the prescribed medication, missed a scheduled follow-up visit and could not be located at home or if they administered additional antimalarial drugs outside the study protocol.

### Laboratory procedures

Thin smears were performed to determine the type of malaria parasite species and thick smears to determine the parasite density and for detection of parasitemia during follow-up. Thick blood smears were stained using 3% Giemsa for 30 min and read by experienced laboratory technologists. All slides were read by two independent microscopists and any discrepant results were reviewed by a tie breaker. Microscopists were blinded to participants’ treatment assignment. In order to blind the microscopists, blood samples were drawn by the study nurse and study numbers were used to label all samples. The study numbers were not known by the microscopists and they did not have access to study data including study databases, participants’ assessments and clinical notes. The study doctors and nurses were aware that laboratory technicians were blinded to treatment assignment and were trained not to reveal the treatment assignments to the microscopists.

Parasite density was calculated by counting the number of asexual parasites (ring stages) per 200 white blood cells (WBCs) or per 500 if the count was less than ten parasites per 200 WBCs, assuming a WBC count of 8000/uL of blood. A smear was considered negative if no parasites were seen after review of 100 high-power fields. Complete blood count and hemoglobin estimation were performed using the Coulter counter, Beckman coulter, Life Science, United States of America.

Filter paper blood samples were collected and subsequently molecular genotyping was conducted for participants with parasitological treatment failure to distinguish re-infection from recrudescence. We used Whatman 3MM Filter paper from Sigma. Molecular genotyping was performed at the Makerere University-University of California San Francisco Molecular Biology laboratory in Mulago, Kampala. Parasite DNA was extracted from filter paper blood samples collected on the day of enrollment and the day of parasitological treatment failure using Chelex 100 Resin extraction (Bio-Rad Laboratories, Hercules, CA) as previously described [15]. Paired samples were genotyped. The surface antigen loci MSP1, MSP2 and GLURP were amplified using previously described primers [16]. Briefly, 2 μL of template DNA was amplified using nested polymerase chain reaction (PCR), with second round primers specific to allelic families: K1, MAD20, and RO33 for msp1, msp2 and the repeat region of glurp [17]. PCR products were stained with ethidium bromide separated by electrophoresis on a 2.5% agarose gel (UltraPure Agarose; Invitrogen, Carlsbad, CA). GelCompar II software (Applied Maths, Sint-Martens-Latem, Belgium) was used to select alleles and estimate the size of PCR products using a standardized approach [18]. Recrudescence was defined as the presence of all matched alleles on day 0 and the day of failure at every locus and reinfection defined as at least one locus showing unmatched alleles.

### Classification of outcomes

To evaluate the 42 day parasitological treatment outcomes following treatment of severe malaria with intravenous AS or intravenous QNN plus ACT (AL or DP); we selected the primary study outcome as parasitological treatment failure unadjusted by genotyping classified as parasitemia detected by thick blood smear during follow-up. This primary outcome was selected because it best represents the treatment outcome measure used in routine clinical care. The secondary outcomes were parasitological treatment failure adjusted by genotyping classified as positive PCR on any follow-up day categorised as reinfection or recrudescence. Adverse events were defined as any medical occurrence post study drug administration. They were graded as mild, moderate, severe and life threatening and their relationship to the study drug was classified as unrelated, possibly, probably or definitely related to study drug.

### Sample size estimation

We estimated the proportion of parasitological failure of 23.6% for intravenous QNN followed by ACT. Assuming a 50% difference in the proportion of patients with parasites at 24 h following start of IV AS, using an alpha of 5%, power of 80%, and adjusting for possible loss to follow up of 10%, a total of 330 patients were needed.

### Statistical analysis

Data were entered and verified using MS ACCESS and analyzed using STATA version 13.1 (STATA Corporation, College Station, TX, USA). Descriptive statistics were used to compare demographic and clinical characteristics among the four study arms. Continuous variables were compared using Wilcoxon test for non-normally distributed data. Categorical variables were compared using Chi-square test. Parasite density was normalized using logarithmic transformation. Intention-to-Treat analysis was used, which included all enrolled participants. Unadjusted treatment failure was classified as a positive blood smear on any of the follow-up days. Adjusted treatment failure was classified as either re-infection or recrudescence based on genotyping. The risk of treatment failure at 28, 35 and 42 days of follow up (unadjusted and adjusted by genotyping) were estimated using the Kaplan-Meier survival method and compared using the Log Rank test. Time at risk was calculated from day one of ACT allocation to date of treatment failure among participants who failed, last day of follow-up for those who did not complete follow-up, or day 42 for the patients who completed 42 days of follow-up. In the analysis for adjusted parasitological outcomes, only recrudescence was considered as true parasitological treatment failure. Safety data from all participants were analyzed. In a sensitivity analysis, we imputed missing genotype data among the treatment failures using multiple imputation with chained equations. Variables included in the imputation model were age, sex, weight, parasite density at baseline visit and treatment arm. Five rounds of imputations were performed. Statistical tests were performed at 5% level of significance and 95% confidence intervals.

## Results

**Fig. 1:**
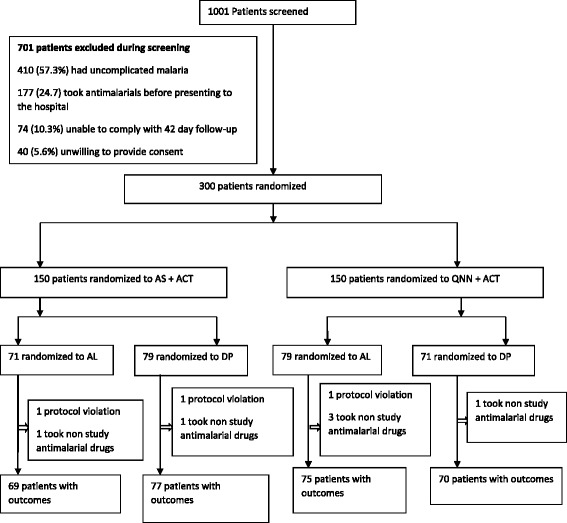
Schematic Diagram of the Trial Profile

We enrolled and followed up 300 participants between January 2012 and March 2013 (Fig. 1). Baseline characteristics were similar across the four treatment arms (Table 1). Nineteen participants (6.3%) did not complete follow-up, of whom; 8 (2.6%) were lost to follow-up, 3 (1%) had protocol violation, 6 (2%) took non study antimalarial drugs and were withdrawn and 2 (0.6%) died on arrival after randomization but before receiving treatment. All the 300 participants were included in the intention to treat analysis. Follow-up outcomes were available for 281 (93.7%) of the 300 participants enrolled, of whom 178 (63.3%) had unadjusted parasitological failure. One participant developed convulsions within 2 h of receipt of the first dose of intravenous AS and died.

**Table 1:** Baseline characteristics of study participants

Characteristic	AS + DP *N* = 79	AS + AL *N* = 71	QNN + DP N = 71	QNN + AL N = 79
Female (%)	36 (45.6)	36 (50.7)	24 (33.8)	37 (46.8)
Age in months^a^	17 (11–26)	16 (10–26)	17 (13–26)	18 (13–26)
Weight (kgs)^a^	9.5 (8–11.5)	9 (8.1–11)	9.8 (8.8–11)	9.2 (8.4–11)
Temperature (degrees Centigrade)^a^	38.8 (37.7–39.5)	39.1 (37.7–39.5)	39.1 (37.3–39.5)	38.6 (37.5–39.6)
Parasite density per uL, log10 copies^a^	4.82 (4.29–5.10)	4.79 (4.38–5.02)	4.72 (4.19–5.00)	4.73 (4.17–5.03)
Complications at admission, n (%)				
Hemoglobin (mg/dL)^a^	9.1 (7.9–10.5)	9.2 (8.4–10.6)	9.3 (8.4–10.4)	9.4 (8.0–10.3)
Total white blood cell count (^a^10^3/^uL)^a^	9.6 (6.9–13.2)	9.2 (7.5–12.3)	9.4 (7.7–12.1)	10.0 (7.0–14.3)
Random blood sugar (mmol/L)^a^	7.3 (6.3–8.3)	6.8 (6.4–8.3)	6.8 (5.7–8.3)	7.4 (6.25–8.25)
History of repeated convulsions n (%)	6 (7.6%)	3 (4.2%)	8 (11.3%)	1 (1.3%)
History of inability to feed	26 (33.0%)	24 (34.0%)	22 (31.0%)	29 (36.7%)
Prostration (extreme weakness)	22 (27.9%)	15 (21.13%)	21 (29.58%)	21 (26.58%)
Hemoglobinuria	0	0	2 (2.8%)	0
Jaundice	2 (2.5%)	0	3 (4.2%)	3 (3.8%)
Severe anemia	0	0	1 (3%)	2 (2.5%)
Respiratory distress	2 (2.5%)	6 (8.5%)	3 (4.2%)	5 (6.3%)
Impaired consciousness	0	0	1 (1.4%)	0
Abnormal bleeding	2 (2.5%)	1 (1.4%)	0	1 (1.3%)
Hypoglycemia	0	0	0	1 (1.3%)

^a^Values are presented as medians (IQR)

### Unadjusted parasitological treatment outcomes

**Fig. 2:**
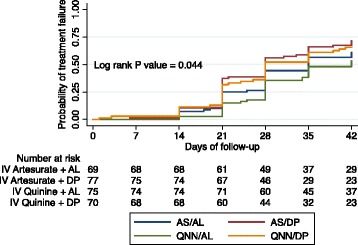
Kaplan-Meier survival curves of the risk of unadjusted treatment failure categorized by ACT arm over 42 days of follow-up compared using the Log Rank test

The median and interquartile range (IQR) for number of days to parasite clearance was significantly lower among participants who received intravenous AS compared with participants who received intravenous QNN (2 (1–2) vs 3 (2–3), *P* < 0.001). During the 42-day follow-up period, 63.3% (178/281) of the participants were classified as having unadjusted parasitological treatment failure; occurring in 41/71 (57.7%, CI 45.4–69.3) of participants who received AS + AL, 53/79 (67.1%, CI 55.6–77.2) of those who received AS +DP, 39/79 (49.4%, CI 37.9–60.8) of those who received QNN + AL and 45/71 (63.4%, CI 51.1–74.5) of those who received QNN + DP. The 42 day risk of unadjusted parasitological treatment failure was 40% lower for participants who received QNN + AL (HR 0.6, 95% CI 0.39–0.9, *p* = 0.014) but not different for those who received QNN + DP (HR 0.8, 95% CI 0.5–1.3, *p* = 0.55) or AS + AL (HR 0.7, 95% CI 0.5–1.1, *p* = 0.16) compared to those who received AS + DP. The risk of getting unadjusted treatment failure was not different across the four study arms (*p* = 0.044) (Fig. 2).

### Adjusted parasitological outcomes

Molecular genotyping was performed for the first 127 (71.3%) of the 178 participants with unadjusted parasitological failures, of whom; 30/127 (23.6%) received AS + AL, 37/127 (29.1%) received AS + DP, 29/127 (22.8%) received QNN + AL, and 31/127 (24.4%) received QNN + DP. Majority (93, 73.2%) had re-infection while 34 (26.7%) had recrudescence as the cause of treatment failure.

Reinfection occurred in 24/71 (33.8%, CI 23.0–46.0) of participants who received AS + AL, 30/79 (37.9%, CI 27.3–49.6) of those who received AS +DP, 21/79 (26.6%, CI 17.3–37.7) of those who received QNN + AL and 18/71 (25.3%, CI 15.8–37.1) of those who received QNN + DP. The risk of getting reinfection was not different across the four study arms (*p* = 0.155).

**Fig. 3:**
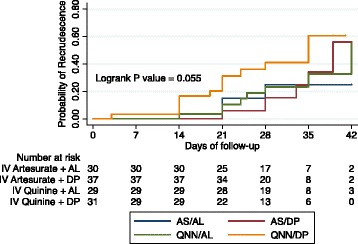
Kaplan-Meier survival curves of the risk of adjusted treatment failure categorized by ACT arm over 42 days of follow-up compared using the Log Rank test

Recrudescence occurred in 6/71 (8.4%, CI 3.2–17.5) of participants who received AS + AL, 7/79 (8.9%, CI 3.6–17.4) of those who received AS +DP, 8/79 (10.1%, CI 4.5–18.9) of those who received QNN + AL and 13/71 (18.3%, CI 10.1–29.3) of those who received QNN + DP. The probability of having recrudescence was highest among patients who received QNN + DP (HR 1.95, 95% CI: 0.78–4.89, *p* = 0.154), followed by QNN + AL (HR 0.95, 95% CI: 0.3–2.6, *p* = 0.93) and AS + AL (HR 0.84, 95% CI: 0.28–2.51, *p* = 0.762) compared to those who received AS + DP. The 42 day risk of recrudescence was not different among the four ACT study arms (*p* = 0.055) (Fig. 3).

In the sensitivity analysis, 41/178 (23.0%) received AS + AL, 53/178 (29.8%) received AS + DP, 39/178 (21.9%) received QNN + AL, and 45/178 (25.3%) received QNN + DP. Majority (124, 69.7%) had re-infection while 54 (30.3%) had recrudescence as the cause of treatment failure. Reinfection occurred in 35/71 (49.3%, CI 37.2–61.4) of participants who received AS + AL, 41/79 (51.9%, CI 40.4–63.3) of those who received AS +DP, 26/79 (32.9%, CI 22.7–44.3) of those who received QNN + AL and 22/71 (31.0%, CI 20.5–43.1) of those who received QNN + DP. The risk of getting reinfection was not different across the four study arms (*p* = 0.155). Recrudescence occurred in 6/71 (8.4%, CI 3.2–17.5) of participants who received AS + AL, 12/79 (15.2%, CI 8.1–25.0) of those who received AS +DP, 13/79 (16.4%, CI 9.1–26.5) of those who received QNN + AL and 23/71 (21.8%, CI 21.8–44.5) of those who received QNN + DP. The risk of getting recrudescence was not different across the four study arms (*p* = 0.055).

### Secondary outcomes

Adverse events occurred commonly and most were of mild to moderate severity and consistent with malaria symptoms. The most common were headache, nausea and vomiting. Among the 281 participants with complete follow-up, 22 (7.8%) experienced severe adverse events, all classified as unrelated to study drugs. Severe adverse events were commonest among participants who received QNN + AL (10/22, 45.4%) followed by AS + DP (5/22, 22.7%), QNN + DP (4/22, 18.1%) and least among those who received AS + AL (3/22, 13.6%). Pneumonia was the commonest severe adverse event occurring in 12/22 (55%) followed by gastro enteritis in 6 (27%) and severe anemia in 4 (18%). All the adverse events were treated and resolved completely except for one participant who experienced repeated convulsions, after receiving intravenous AS and died within 2 h post drug administration.

## Discussion

We evaluated the 42-day parasitological treatment outcomes and safety following treatment of children with severe malaria using intravenous AS or intravenous QNN plus ACT (AL or DP) in Tororo District Hospital in Eastern Uganda. Participants who received intravenous AS cleared malaria parasites significantly faster than those who received intravenous QNN, which is consistent with data from previous large trials [3, 5].

There was low mortality among our study participants. The risk of death for patients with severe malaria is high and increases with delayed initiation of treatment and in presence of multiple complications. Risk of death is also dependent on the patient’s age, background immunity, pre-morbid and concomitant diseases. We did not study time intervals between start of symptoms and presentation to hospital or receipt of antimalarial drug. Our previous study demonstrated longer duration of illness before receiving antimalarial medication among children presenting to hospital with severe malaria compared to those who had uncomplicated malaria [19]. It is possible that the participants who died experienced delay in presentation to hospital for various reasons such as challenges in transportation and poor health seeking behavior of the care takers.

Both AL and DP were effective at clearing residual parasites during the 42-day follow-up period. Majority of our study participants classified as treatment failure actually suffered re-infection with malaria parasites during the 42-day follow-up period, with higher reinfection rates among participants who received AS + ACT compared to QNN + ACT regimens. Our findings are consistent with previous data from similar high malaria transmission settings which demonstrated high rates of re-infection with malaria parasites after initial treatment [20, 21]. Our finding of the highest re-infection rates among participants who received AS + DP although not statistically significant, is surprising and difficult to explain since piperaquine which is the longer acting partner drug in the DP combination is known to have a long half-life of about 35 days compared to lumefantrine’s half-life of 28 days, thus is expected to offer longer post treatment prophylactic effect [12, 21, 22]. Previous studies reported lower malaria re-infection rates following treatment with DP compared to AL [20, 21], our findings are possibly limited by the small study numbers for that comparison. Recurrent malaria infections have been associated with higher risk for development of symptomatic malaria disease than recrudescent parasitemia [21, 23]. The high malaria re-infection rate is particularly significant in patients who have suffered severe malaria attacks since re-infection is likely to result into recurrent attacks of severe malaria with co-morbidity such as anaemia and resultant negative health and social-economic impact. Although we did not evaluate the information given to patients at discharge; we emphasize the importance of education of patients and caretakers on enhancing malaria prevention.

The high rate of malaria re-infection in our study population, calls for strengthening of national efforts in malaria prevention, with particular emphasis on high malaria transmission settings. Interventions such as long lasting insecticide treated bed nets, indoor residual spraying with insecticides, malaria chemoprophylaxis for high risk groups, are highly effective in reducing malaria morbidity [24] and have been rolled out in this study area, however, further sensitization of communities and behavior change are needed. The malaria vaccine would seemingly provide a more substantial solution to this overwhelming burden of malaria. The RTS, S/AS01 malaria vaccine was evaluated in a large phase three trial and received positive regulatory assessment, however, due to unresolved uncertainties, the WHO made recommendations for further evaluation of this vaccine to address gaps in knowledge before wider country level introduction [24].

It is important to understand why some children living in malaria endemic areas are at higher risk for development of severe malaria and recurrent malaria attacks than others living in the same areas. Several hypothesis for host susceptibility to severe malaria and re-infection have been suggested and some are under study [19, 25]. These need to be evaluated and addressed. Additional measures to reduce malaria morbidity include use of Intermittent Preventive Treatment of malaria in infants (IPTi) using sulphadoxine-pyrimethamine (SP). This is recommended for areas with low prevalence of key polymorphisms that mediate diminished parasite response to SP. In Uganda; IPTi with SP is not recommended because the prevalence of these polymorphisms is consistently greater that 50% across the country [26]. A study in an area with intense malaria transmission in Malawi demonstrated that monthly chemoprophylaxis with AL post treatment for severe malarial anaemia reduced rates of readmittance to hospital for severe anaemia or malaria [27]. Additional studies on IPT for school children living in such high malaria transmission settings demonstrated acceptable protective efficacy against clinical malaria, parasitaemia and anaemia with use of either DP, SP + AS or Amodiaquine + AS. Monthly DP provided the best protective efficacy [28]. Further studies to investigate the acceptability, cost effectiveness and long term safety of these IPT regimens are needed.

We demonstrated low levels of recrudescence among our study participants with no difference in the 42-day risk of recrudescence among the four ACT study arms. Among the few participants in whom recrudescence was demonstrated; the highest probability of recrudescence was among participants who received the combination of QNN + DP followed by QNN + AL, although this finding was not statistically significant possibly due to the limitation of the small sample size for this comparison. Our study was also limited by the genotyping method used which is known to have low resolution. The long acting drug should ideally mop up all residual parasites to prevent recrudescence while also preventing re-infection. It is possible that the QNN + ACT regimens predisposed participants to higher rates of recrudescence due to slower initial clearance of parasite biomass by intravenous QNN compared to intravenous AS. This finding supports the current recommendation to use intravenous AS in preference to intravenous QNN for initial treatment for severe malaria and calls for improved availability and accessibility to intravenous AS especially in the high transmission areas.

The study participants’ clinical characteristics did not differ among study groups with the commonest forms of severe malaria documented to be; inability to feed and prostration. Our study participants did not present with very severe forms of malaria, a finding that is consistent with data from previous studies which demonstrated lower risk of very severe forms of malaria among individuals living in areas with high malaria transmission most likely due to development of partial immunity from recurrent malaria infections early in life [29]. Individuals living in malaria endemic regions are frequently exposed to malaria infection and develop partial immunity which helps to clear parasites from circulation upon subsequent infection preventing the development of very severe forms of malaria.

## Conclusions

We observed adequate initial treatment outcomes followed by very high rates of malaria re-infection post severe malaria treatment with intravenous AS or intravenous QNN followed by ACT (AL or DP) in a high malaria transmission setting. The impact of recurrent antimalarial treatment on the long term efficacy of antimalarial regimens needs to be investigated and surveillance mechanisms for resistance markers established since recurrent malaria infections are likely to be exposed to sub-therapeutic drug concentrations. More strategies for prevention of recurrent malaria infections in the most at risk populations are needed.

## Abbreviations

ACT: Artemisinin based combination therapy
AL: Artemether-lumefantrine
AS: rtesunate
DHA: Dihydroartemisinin
DP: Dihydroartemisinin-piperaquine
PCR: Polymerase chain reaction
PQP: Piperaquine
QNN: Quinine
WHO: World Health Organsistaion
